# Antiangiogenic Medications Impede the Oral Mucosal Microcirculation and Interfere with Oral Wound Healing: A Complication Deserving of Attention

**DOI:** 10.1002/mco2.70279

**Published:** 2025-07-11

**Authors:** Hongyuan Huang, Ning Zhao, Jianhua Zhu, Qingxiang Li, Qiao Qiao, Yuanning Yang, Ying Zhou, Chuanbin Guo, Yuxing Guo

**Affiliations:** ^1^ Department of Oral and Maxillofacial Surgery Peking University School and Hospital of Stomatology Beijing PR China; ^2^ National Clinical Research Center for Oral Diseases Beijing PR China; ^3^ National Engineering Laboratory for Digital and Material Technology of Stomatology Beijing PR China; ^4^ Beijing Key Laboratory of Digital Stomatology Peking University School and Hospital of Stomatology Beijing PR China

**Keywords:** hand‐held vital microscope, medication‐related osteonecrosis of the jaw, microcirculation, wound healing

## Abstract

Long‐term antiangiogenic therapy may be linked to medication‐related osteonecrosis of the jaw (MRONJ), complicating surgical treatment due to impaired postoperative wound healing. However, the mechanisms underlying these healing difficulties remain unclear. This bidirectional cohort study explored the impact of antiangiogenic medications (AGM) in combination with antiresorptive medications (ARM) on oral mucosal microcirculation and its relationship with surgical outcomes in MRONJ patients. A total of 30 patients (15 using ARM and 15 using both ARM and AGM) and 15 healthy volunteers undergoing surgery were included. A handheld vital microscope (HVM) was utilized to assess oral mucosal microcirculation. The results showed that patients taking both AGM and ARM had reduced microvascular density and more stagnant microcirculation than patients taking ARM alone and healthy volunteers. Additionally, impaired microcirculation was also statistically linked to poorer surgical prognosis in MRONJ patients receiving AGM&ARM with lower microcirculation parameters. This study highlights the potential adverse effect of AGM on oral mucosal microcirculation, contributing to impaired wound healing after surgery in MRONJ patients. This study provides new evidence for the vascular mechanisms involved in healing difficulties in MRONJ and supports the hypothesis that AGM plays a detrimental role in oral surgical recovery.

## Graphical abstract

The combined use of antiangiogenic medication and antiresorptive medication aggravates the state of oral mucosal microcirculation. Antiangiogenic agents could affect postoperative wound healing in antiresorptive‐drug‐based MRONJ patients by the way of impairing the oral mucosa microcirculation. The HVM device provides an non‐invasive and accurate way to detect oral mucosal microcirculation and therefore predict surgical prognosis.

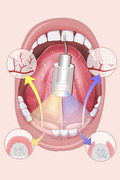

## 1 Introduction

Neoangiogenesis, the formation of new blood vessels, is crucial in supplying essential nutrients and oxygen to tumor cells, driving cancer proliferation, invasion, and metastasis [1, 2]. Therefore, antiangiogenic therapies have become an important direction for cancer treatment [1, 3, 4, 5]. These medications aim to inhibit the growth and spread of tumors by weakening or blocking their vascular networks, thus depriving tumor cells of nutrients and oxygen supply. Their clinical effectiveness has also been validated across multiple cancer types [6, 7, 8, 9, 10]. Antiangiogenic medications (AGMs) fall into two main categories: small‐molecule tyrosine kinase inhibitors (TKIs) and monoclonal antibodies [11]. TKIs such as sunitinib and cabozantinib act directly on intracellular tyrosine kinases to inhibit the vascular endothelial growth factor (VEGF) signaling pathway. Monoclonal antibodies, such as bevacizumab and ramucirumab, specifically bind to VEGF or its receptors, blocking their interaction with cell surface receptors and directly inhibiting angiogenic signaling. Due to the critical role of VEGF in maintaining vascular‐related homeostasis, blocking its function not only inhibits tumor growth but may also trigger a series of adverse effects related to the circulatory system, kidneys, and wound healing [12, 13, 14, 15, 16].

The combined use of antiangiogenic medications and antiresorptive medications (ARM) is particularly common in the treatment of cancer patients with bone metastases, which may lead to a potentially serious oral complication known as medication‐related osteonecrosis of the jaw (MRONJ) [17, 18, 19]. MRONJ presents with persistently impaired healing of the intraoral mucosa with persistent pus overflow and exposure of the lesion jaws [20]. Although antiresorptive therapy coupled with local infection or trauma is necessary and sufficient to induce MRONJ, angiogenesis inhibition seems to be implicated in the pathophysiology of this disease [21, 22, 23]. Several studies have reported that a combination of antiresorptive and antiangiogenic therapies is associated with a higher MRONJ prevalence and poor prognosis after surgical treatment [18, 19, 24, 25], suggesting that AGM might be the major factor in oral mucosal wound healing impairment. Wound healing is a cascading biological process divided into four distinct but overlapping phases: hemostasis and coagulation, inflammation, cell proliferation, tissue remodeling, and maturation [26]. Given that angiogenesis has a crucial role in the proliferation phase of wound healing, it is important to examine the factors that affect oral mucosal microcirculation in MRONJ patients under various medication regimens (ARM or AGM) and how these factors impact postoperative wound healing.

Immunochemistry is commonly used to assess the number of microvessels. However, this method requires mucosal specimen excision, which is invasive and unsuitable for the clinical evaluation of postsurgical healing. Over the years, a hand‐held vital microscope (HVM) has been proposed based on sidestream dark‐field imaging (SDF), whereby illumination is provided by surrounding a central light guide by concentrically placed light‐emitting diodes to create dark‐field illumination. Light from the HVM probe at a central wavelength of 530 nm can be absorbed by hemoglobin in the red blood cells, leading to an image where red blood cells are imaged as dark‐moving globules against a grayish background [27, 28]. HVM offers convenient, efficient, and precise detection of mucosal microcirculation.

In this study, the HVM device was employed to assess the mucosal microcirculation in patients with MRONJ, utilizing the two most prevalent medication regimens (ARM or ARM+AGM), with findings confirmed through immunohistochemical staining. The relationship between different microcirculation statuses and medication regimens and the relationship between microcirculation status and wound healing after surgery were analyzed.

## 2 Results

### 2.1 Optimal Blood Pressure for Monitoring Sublingual Microcirculation

**FIGURE 1:**
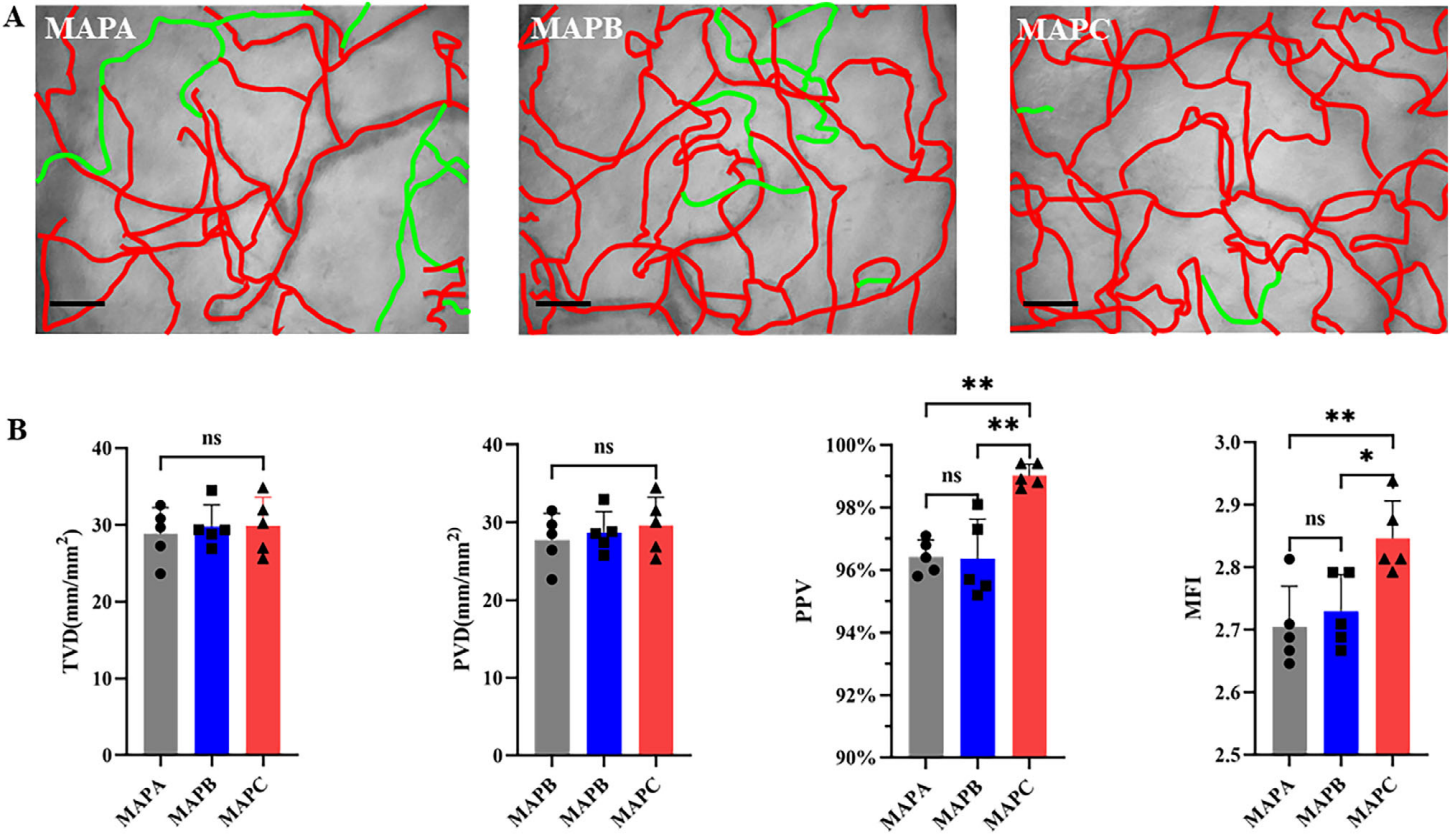
Optimal blood pressure for monitoring sublingual microcirculation. (A) Representative images of sublingual microcirculation acquired by HVM. (B) One‐way analysis of variance was used to compare TVD, PVD, PPV, and MFI among three blood pressure ranges. Data are presented as mean ± SD followed by a significant test. MAPA, mean arterial pressure A (75∼85 mmHg); MAPB, mean arterial pressure B (85∼95 mmHg); MAPC, mean arterial pressure C (95∼105 mmHg); TVD, total vessel density; PVD, perfused vessel density; PPV, proportion of perfused vessels; MFI, microvascular flow index. Scale bar =100 µm. ns, *p* > 0.05; *, *p* < 0.05; **, *p* < 0.01.

Five healthy subjects were enrolled, and sublingual microcirculation videos were recorded using the method mentioned in section 4.4. After analyzing images of the sublingual microcirculation at three different blood pressure intervals (Figure 1A; Video S1), there was no statistical difference among the three blood pressure intervals for TVD (TVD_MAPA_ = 28.79 ± 3.465 mm/mm^2^; TVD** **_MAPB_ = 29.78 ± 2.826 mm/mm^2^; TVD_MAPC_ = 29.89 ± 3.716 mm/mm^2^, *p* = 0.8532) and PVD (PVD_MAPA_ = 27.76 ± 3.407; PVD _MAPB_ = 28.69 ± 2.678; PVD_MAPC_ = 29.39 ± 3.646, *p* = 0.6854). However, PPV (PPV_MAPA_ = 96.42 ± 0.540%; PPV_MAPB_ = 96.36 ± 1.268%; PPV_MAPC_ = 99.02 ± 0.36%, *p* = 0.0003), and MFI (MFI_MAPA_ = 2.705 ± 0.065; MFI_MAPB_ = 2.730 ± 0.059; MFI_MAPC_ = 2.846 ± 0.060, *p* = 0.0074) values were statistically higher when the blood pressure was MAPC versus MAPA or MAPB and was very close to the maximum values (Figure 1B).

### 2.2 Antiangiogenic Medication Aggravates the State of Oral Mucosal Microcirculation in MRONJ Patients Treated With ARM

**FIGURE 2:**
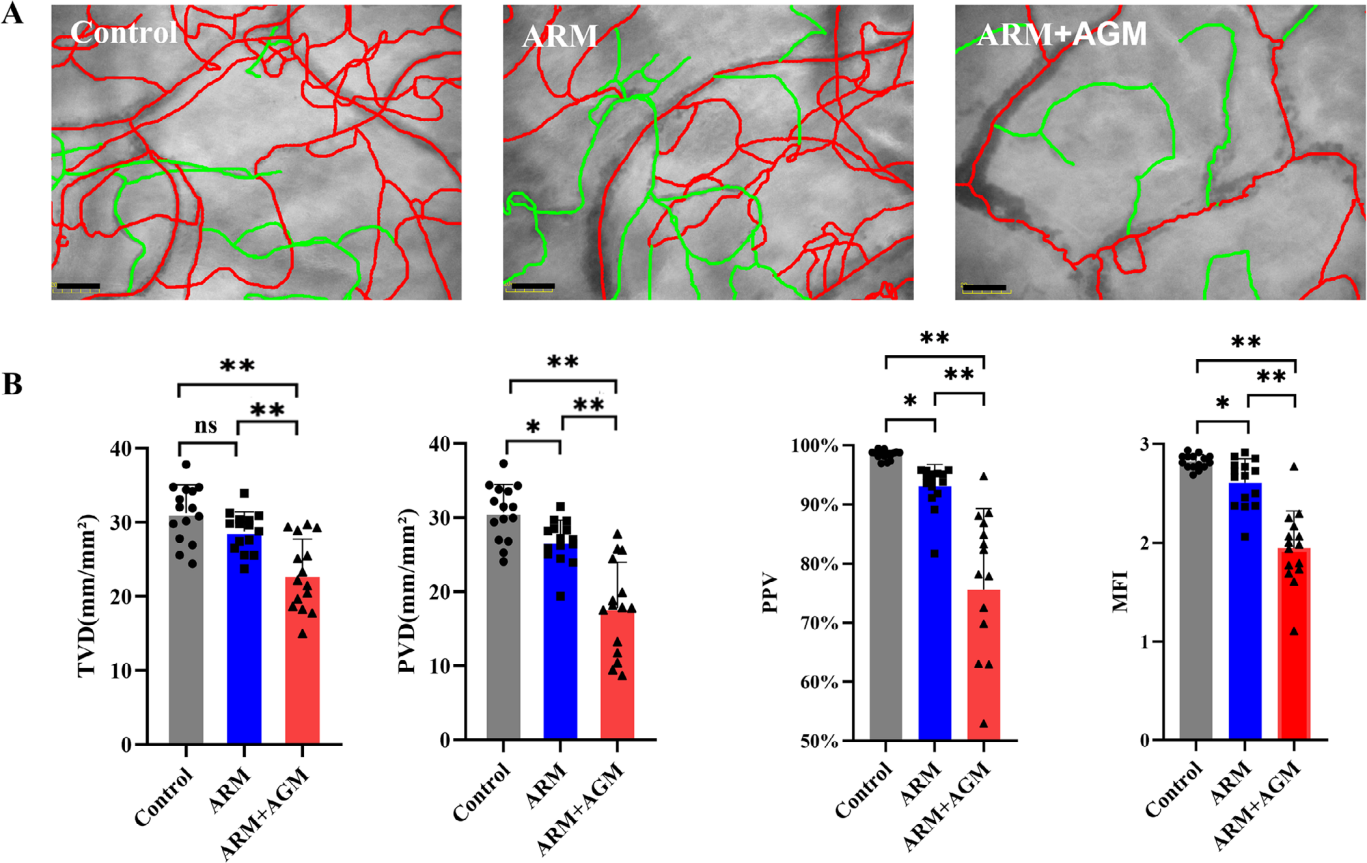
Antiangiogenic medication aggravates oral mucosal microcirculation in MRONJ patients treated with ARMs. (A) Representative images of sublingual microcirculation from three groups (15 patients in the ARM group, 15 cases in the ARM+AGM group, and 15 healthy subjects) acquired by HVM. A red curve represents perfused microvessels; a green curve represents sluggishly flowing or blocked microvessels. (B) One‐way analysis of variance was used to compare TVD, PVD, PPV, and MFI among three groups. Data are presented as mean ± SD followed by a significant test. Scale bar=100 µm. ARM, antiresorptive medication; AGM, antiangiogenic medication. ns, *p* > 0.05; *, *p* < 0.05; **, *p* < 0.01.

A total of 30 MRONJ patients, 15 in the ARM group and 15 in the ARM+AGM group, as well as 15 healthy subjects (control group), were assessed. No differences in demographic data (mean age and sex distribution) were observed between the groups (all *p *> 0.05, Table S1). Sublingual microcirculation videos of all 30 patients with MRONJ and 15 healthy subjects were recorded (Figure 2A; Video S2). ARM+AGM group had the lowest value of TVD compared with the control group and ARM group (TVD_control_ = 31.24±3.81 mm/mm^2^, TVD_ARM_ = 28.75 ± 4.34 mm/mm^2^, TVD _ARM+AGM_  = 22.94 ± 4.82 mm/mm^2^). The difference between the control group and the ARM+AGM group (*p *< 0.0001), as well as between the ARM and ARM+AGM groups (*p *= 0.0005), was significant, whereas there was no significant difference between the control and ARM groups (*p *= 0.194). ARM+AGM group also had the significantly lowest value of PVD, PPV, and MFI among three groups (PVD_Control_ = 30.73 ± 3.75 mm/mm^2^, PVD_ARM_ = 26.79 ± 2.90 mm/mm^2^, PVD_ARM+AGM_ = 17.78 ± 6.21 mm/mm^2^; PPV_Control_ = 98.37 ± 0.77%, PPV_ARM_ = 93.05 ± 3.70%, PPV_ARM+AGM_ = 75.61 ± 13.63%; MFI_Control_ = 2.82 ± 0.07, MFI_ARM_ = 2.61 ± 0.24, MFI_ARM+AGM_ = 1.94 ± 0.37). The values of PVD, PPV, and MFI were also significantly lower in the ARM+AGM group than in the control or ARM group (all *p* < 0.05) (Figure 2B). While the PVD, PPV, and MFI values in the ARM group were also significantly lower than those in the control group (all *p* < 0.05).

### 2.3 The Oral Mucosal Microcirculation Measurements Detected by a Noninvasive HVM Device Were Partially Confirmed with the Immunohistochemistry Staining Results

**FIGURE 3:**
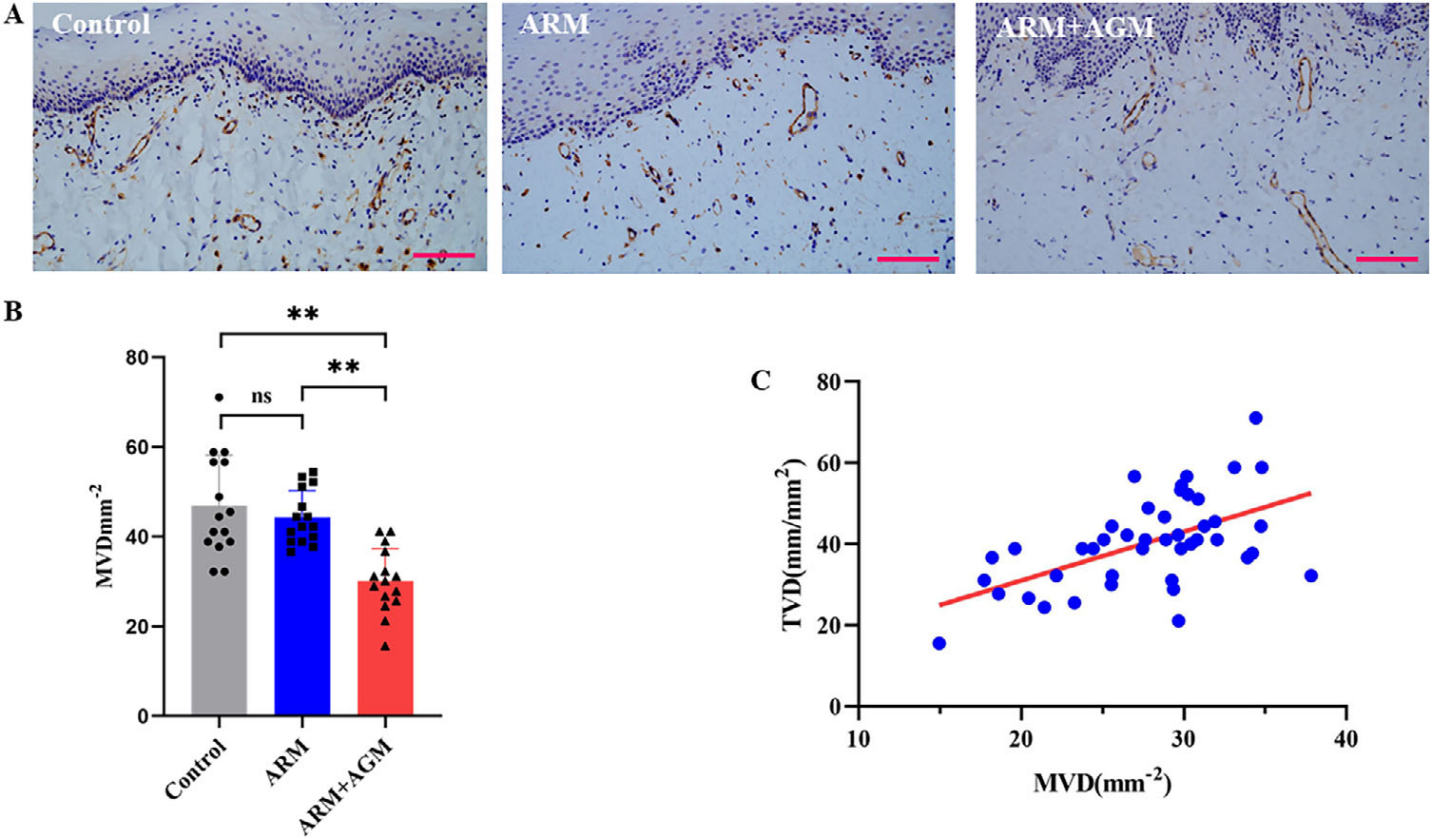
The changes in oral mucosal microcirculation of MRONJ patients detected by HVM were partly confirmed by immunohistochemistry staining. (A) Representative immunohistochemistry images of CD31‐positive microvessels in oral mucosa from three groups (15 patients in the ARM group, 15 cases in ARM+AGM, and 15 healthy subjects). (B) CD31‐positive microvessels for each subject were counted. Data were presented as mean ± SD followed by a significant test. (C) Linear regression was used to analyze the relationship between TVD and MVD in all subjects. The red line represents the fitted regression line for the TVD and MVD (Y=1.207X+6.899). ARMs, antiresorptive medications; AGMs, antiangiogenic medications; MVD, microvessel density; TVD, total vessel density. Scale bar = 100 µm. ns, *p* > 0.05; **, *p* < 0.01.

Oral mucosal specimens were collected to validate the reliability of the sidestream dark‐field imaging technique from the perspective of vascular density based on the IHC staining data (Figure 3A shows a representative example). The microvessel density, assessed by CD31 staining on endothelial cells, was the highest in the control group (microvessel density [MVD] = 46.89 ± 11.29 mm^−2^), followed by the ARM group (MVD = 44.30 ± 5.95 mm^2^) and the lowest in the ARM+AGM group (MVD = 30.15 ± 7.24 mm^2^). The differences in microvessel density between the control group and ARM+AGM group (*p *< 0.001) as well as between the ARMs and ARM+AGM groups (*p* = 0.0003) were significant, while there was no significant difference between the control group and ARMs group (Figure 3B). We also found a positive correlation between the TVD value of noninvasive detection of HMV devices and the MVD value of IHC staining statistics (*r* ^2^ = 0.3075, *p *< 0.0001; Figure 3C).

### 2.4 The Impairment of Oral Mucosal Microcirculation Is Associated With Poor Healing in MRONJ Patients After the Surgical Treatment

We further analyzed the correlation between surgical prognosis and oral mucosal microcirculation in patients with MRONJ. As shown in Table 1, ARM+AGM patients had an advanced MRONJ stage compared with those in the ARM group (*p *= 0.04). In addition, after 6 months of follow‐up, the wound healing rate of patients in the ARM group was significantly higher than that in the ARM+AGM group (ARM healing rate = 86.67%; ARM+AGM healing rate = 40%). At the same time, we found that the TVD, PVD, PPV, and MFI of patients in the unhealing group were worse than those in the healing group (TVD_healing group_ = 29.41 ± 2.83 mm/mm^2^, TVD_unhealing group_ = 21.43 ± 4.40 mm/mm^2^; PVD_healing group_ = 26.09 ± 3.36 mm/mm^2^, PVD_unhealing group_ = 15.71 ± 5.59 mm/mm^2^; PPV_healing group_ = 91.64 ± 5.13%, PPV_unhealing group_ = 71.72 ± 13.54%; MFI_healing group_ = 2.5 ± 0.33, MFI_unhealing group_ = 1.89 ± 0.39; all *p *< 0.01) (Table S2).

**TABLE 1:** Basic information on 30 MRONJ patients divided depending on their drug history.

	ARM(*n* = 15)	ARM+AGM(*n* = 15)	*p‐*value
Primary disease			NA
Lung cancer	5	9	
Kidney cancer	0	5	
Breast cancer	5	1	
Prostate cancer	5	0	
Duration of antiresorptive therapy in months ± SD	26.87 ± 11.46	19.60 ± 7.23	0.03*
MRONJ stage			0.04*
Stage 2	14	8	
Stage 3	1	7	
MRONJ location			0.25
Maxilla	3	7	
Mandible	12	8	
Treatment outcome			0.02*
Healing	13	6	
Unhealing	2	9	

*Note*: 30 MRONJ patients with MRONJ were enrolled in our study, including 15 patients taking ARMs alone and 15 patients taking both ARMs and AGMs. Compared with the ARM+AGM group, patients in the ARM group received a longer duration of antiresorptive therapy and had more serious symptoms and poorer prognosis.

Abbreviations: ARM, antiresorptive medication; AGM, antiangiogenic medication.

### 2.5 Antiangiogenic Agents Affect Wound Healing in MRONJ Patients Treated with ARMs and Who Underwent Surgery

**FIGURE 4:**
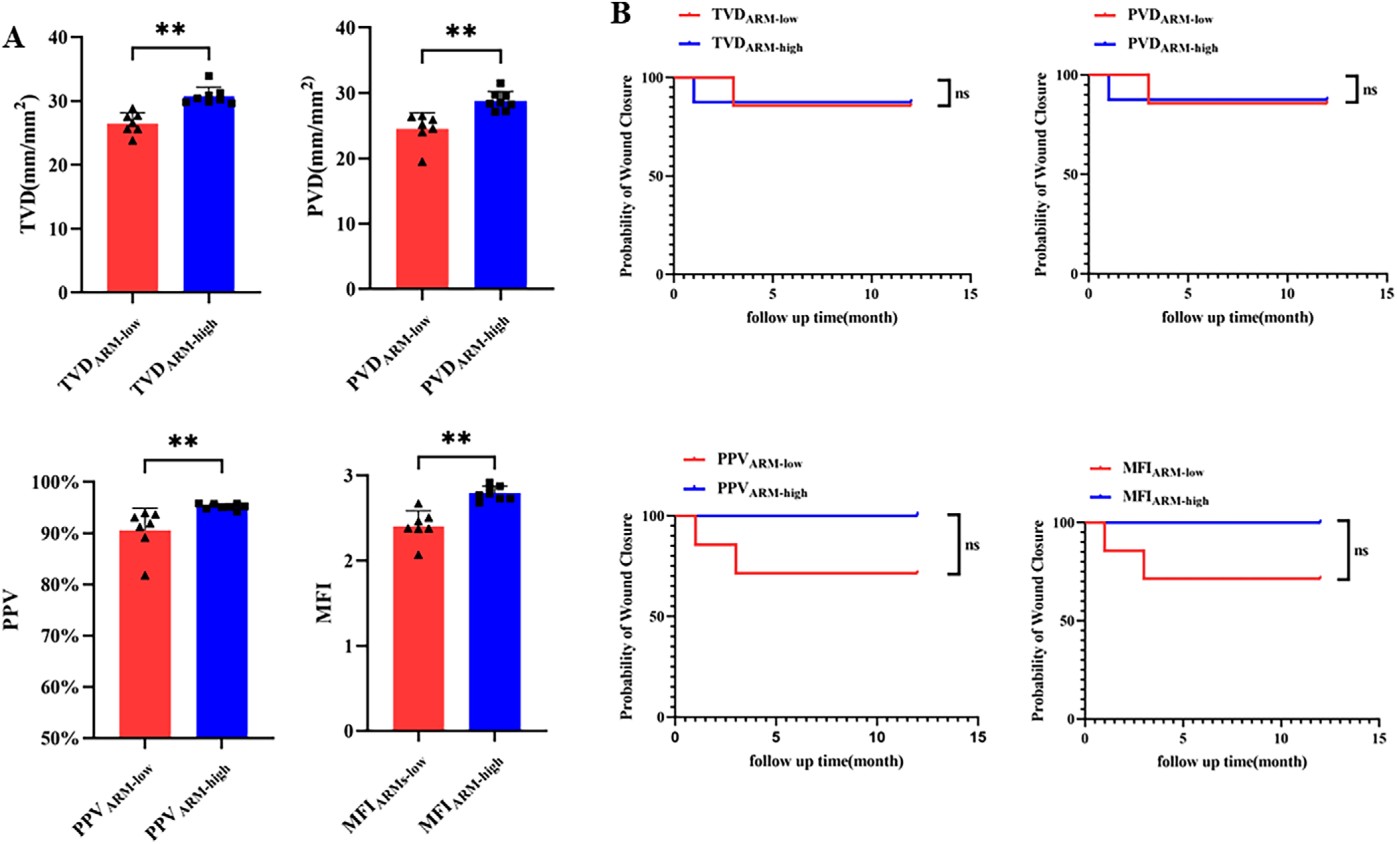
The effect of antiresorptive drugs showed no significant effect on surgical treatment in MRONJ patients. (A) A two‐sided unpaired t‐test was used to compare TVD, PVD, PPV, and MFI between the “high” subgroup and the “low” subgroup in the ARM group. Data were presented as mean ± SD followed by a significant test. (B) Cox regression analysis was used to compare the surgical treatment outcome of MRONJ patients divided according to the values of TVD, PVD, PPV, and MFI in the ARM group. ARMs, antiresorptive medications; TVD, total vessel density; PVD, perfused vessel density; PPV, proportion of perfused vessels; MFI, microvascular flow index. ns, *p* > 0.05; **, *p* < 0.01.

We compared the four parameters of patients with the same medication regimen (ARM alone or ARM+AGM) according to their levels and average values; if the value of one microcirculation parameter was greater than or equal to the median value of the ARM group or ARM+AGM group patients, it was assigned to the “high” subgroup; otherwise, it was assigned to the “low” subgroup. Briefly, there was a statistical difference in microcirculation parameters between the low and the high subgroup when evaluating patients in the ARM group (TVD_ARM‐low_ = 26.47 ± 1.67 mm/mm^2^, TVD_ARM‐high_ = 30.75 ± 1.39 mm/mm^2^; PVD_ARM‐low_ = 24.52 ± 4.25 mm/mm^2^, PVD_ARM‐high_ = 28.77 ± 0.92 mm/mm^2^; PPV_ARM‐low_ = 90.59 ± 4.25%, PPV_ARM‐high_ = 92.50 ± 0.54%; MFI_ARM‐low_ = 2.40 ± 0.18, MFI_ARM‐high_ = 2.79 ± 0.08; Figure 4A), but no difference in the surgical outcomes between subgroups (Figure 4B; Table S3).

**FIGURE 5:**
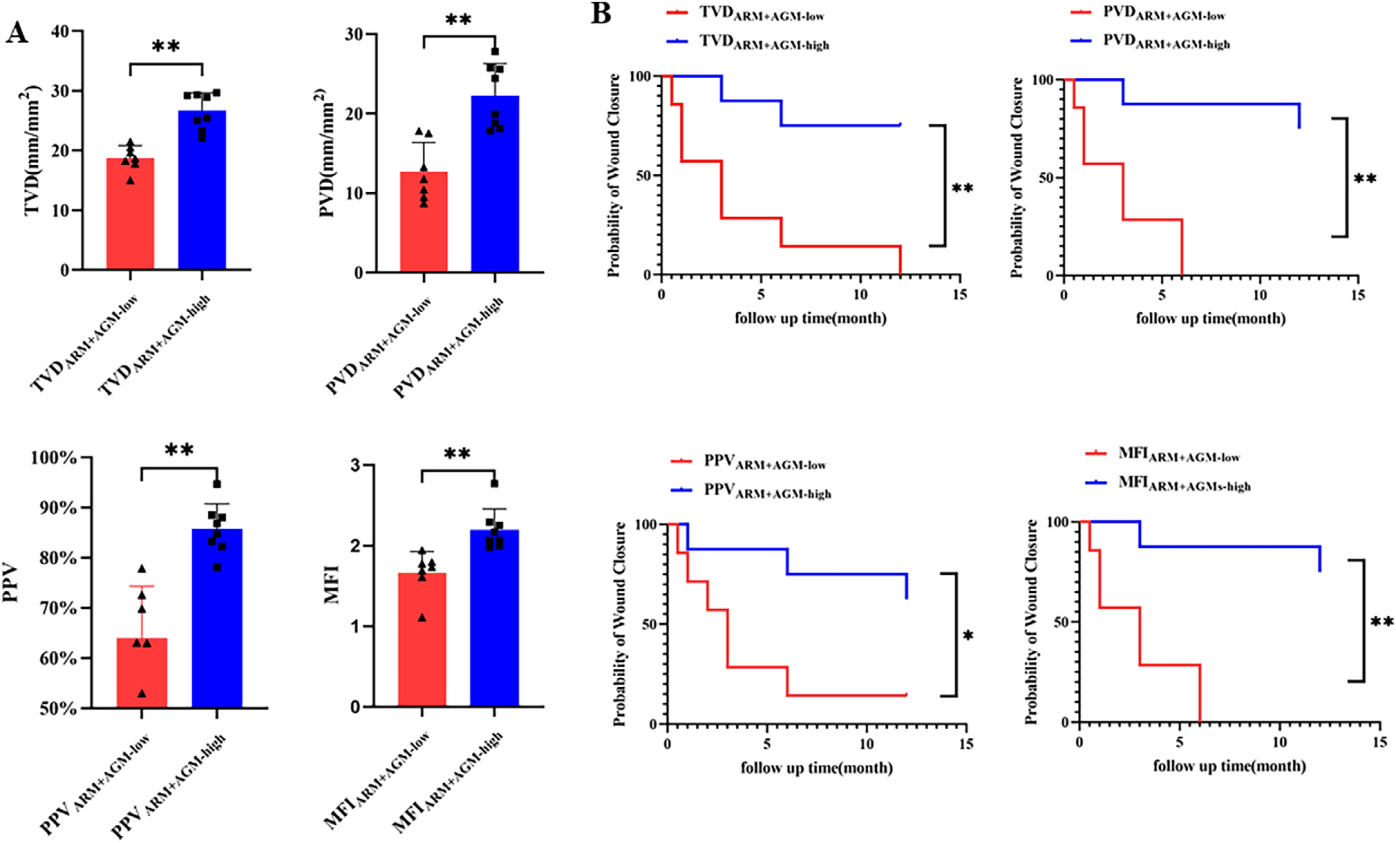
Antiangiogenic agents affect wound healing after surgery in MRONJ patients treated with ARMs. (A) Two‐sided unpaired *t*‐tests were used to compare TVD, PVD, PPV, and MFI between the “high” subgroup and the “low” subgroup in the ARM+AGM group. Data were presented as mean ± SD followed by a significant test. (B) Cox regression analysis was used to compare the surgical treatment outcome of MRONJ patients divided according to the values of TVD, PVD, PPV, and MFI in the ARM+AGM group. TVD, total vessel density; PVD, perfused vessel density; PPV, the proportion of perfused vessels; MFI, microvascular flow index; ARMs, antiresorptive medications; AGMs, antiangiogenic medications. *, *p* < 0.05; **, *p* < 0.01.

In the ARM+AGM group, there was a statistical difference in microcirculation parameters between the “low” subgroup and the “high” subgroup (TVD_ARM+AGM‐low_ = 18.72 ± 2.11 mm/mm^2^, TVD_ARM+AGM‐high_ = 26.64 ± 3.02 mm/mm^2^; PVD_ARM+AGM‐low_ = 12.66 ± 3.71 mm/mm^2^, PVD_ARM+AGM‐high_ = 22.27 ± 4.01 mm/mm^2^; PPV_ARM+AGM‐low_ = 63.95 ± 10.41%, PPV_ARM+AGM‐high_ = 85.80 ± 4.95%; MFI_ARMs+AGMs‐low_ = 1.66 ± 0.27, MFI_ARM+AGM‐high_ = 2.20 ± 0.26; Figure 5A; Video S3). In addition, the results of the univariate Cox regression suggested that patients in “high” subgroups of TVD, PVD, PPV, and MFI had statistically better prognoses than “low” subgroups (Figure 5B). Multifactorial Cox regression results also showed that in the ARM+AGM group, the prognosis of the high TVD and high PVD subgroups was significantly better than that of the low TVD and low PVD subgroups, respectively (Tables 2 and 3). In contrast, in the PPV and MFI subgroups, factors including microcirculation parameters, MRONJ stage, duration of antiresorptive therapy, and MRONJ locations had no significant effect on prognosis (Tables S4 and S5).

**TABLE 2:** The relationship between the study variables with wound healing using multifactorial Cox regression analysis in TVD subgroup of ARM+AGM group (*n* = 15).

Variable	Hazard ratio	95% CI	*p‐*value
TVD (low vs. high)	8.496	1.650–43.737	0.010*
MRONJ stage (stage II vs. stage III)	4.238	0.476–37.739	0.196
Duration of antiresorptive therapy (<median) versus (>median)	0.783	0.171–3.595	0.753
MRONJ location (maxilla vs. mandible)	1.446	0.188‐11.138	0.723

*Note*: Duration of antiresorptive therapy and MRONJ stage with *p* < 0.05 in univariate analyses are selected for further multivariate.

Abbreviations: CI, confidence interval; TVD, total vessel density.

**TABLE 3:** The relationship between the study variables with wound healing using multifactorial Cox regression analysis in PVD subgroup of ARM+AGM group (*n* = 15).

Variable	Hazard ratio	95% CI	*p‐*value
PVD (low vs. high)	247.894	7.356–8354.131	0.002**
MRONJ stage (stage II vs. stage III)	0.180	0.017–1.913	0.155
Duration of antiresorptive therapy (<median) vs. (>median)	0.058	0.005–0.689	0.024*
MRONJ location (maxilla vs. mandible)	0.348	0.056–2.142	0.255

*Note*: Duration of antiresorptive therapy and MRONJ stage with *p* < 0.05 in univariate analyses are selected for further multivariate.

Abbreviations: CI, confidence interval; PVD, perfused vessel density.

## 3 Discussion

Due to the important role of angiogenesis in tumor development and metastasis, antiangiogenic therapy has become an important option in oncological treatment. Combination therapy, including ARM and AGM, is commonly used for patients with bone metastasis. However, this treatment is associated with certain side effects and complications such as MRONJ [29]. MRONJ manifests as persistent impaired healing of the intraoral mucosa, chronic purulent drainage, and exposure of the lesion jaws. Clinical studies have shown that patients with MRONJ who undergo surgical treatment also tend to have unhealed wounds, especially those with a history of antiangiogenic medication combinations [29, 30, 31].

Wound repair is a complex process consisting of four separate but closely related phases, that is, hemostasis, inflammation, proliferation, and dermal remodeling. New blood vessels are created during angiogenesis to meet the metabolic demands of the highly proliferative healing tissue, which has an essential role in microvascular homeostasis [26, 32]. The release of VEGF and other factors prompts microvascular endothelial cells to proliferate and migrate into the wound bed, sprouting new vessels, fusing with others to form stable, tubular networks, and prevents endothelial cell apoptosis by upregulating anti‐apoptotic proteins such as BCL‐2 [33, 34]. In this study, we discovered that patients who received a combination of ARM and AGM, which targets the VEGF pathway, had a more advanced MRONJ stage and a poorer prognosis than those who received ARM alone. We hypothesized that the severe suppression of antiangiogenic activity in the oral mucosa may be a reason for the more severe clinical symptoms and the failed wound healing in MRONJ patients.

Mucosal angiogenic activity dramatically affects the local microvessel density and mucosal microcirculation. In this study, the microcirculation status of the oral mucosa in patients with MRONJ and age‐matched healthy volunteers was assessed using HVM, a hand‐held instrument that visualizes submucosal microvessels, which was confirmed using immunohistochemistry. When analyzing sublingual microcirculation images obtained from HVM, our results indicate that there is no significant difference in the TVD index, which represents overall microvascular density, between the ARM group and the control group. This finding is consistent with subsequent CD31 staining results. However, indices reflecting microcirculatory perfusion status, such as PVD, PPV, and MFI, were significantly lower in the ARM group compared with the control group. The study by Kosach German et al. [35] demonstrated that the use of ARM impairs the dilatory function of nailfold and gingival microvessels in MRONJ patients, further suggesting the detrimental effects of ARM on microcirculatory function. It may be because ARMs such as bisphosphate lead to a transient impairment of microvascular diastolic function but do not lead to a sustained decrease in microvessel density, owing to rapid drug metabolism or rapid drug deposition in the bone [36]. Bastos et al. [37], however, showed that mucosal microvessel density was significantly lower in the marginal region of MRONJ necrotic lesions compared with healthy controls. However, this study included only four MRONJ patients, which may introduce potential bias. In contrast, MRONJ patients receiving both antiresorptive and antiangiogenic therapies showed decreased oral mucosal microvascular density (TVD) and function (PVD, PPV, MFI), implying that it not only impairs microvascular function but also reduces local microvascular density. This is further confirmed by the CD31 immunohistochemistry results.

Successful MRONJ surgery is determined by primary wound healing and the preservation of complete mucosal coverage [38, 39]. Because angiogenesis has an important role in wound healing, and microcirculation status in the oral mucosa may indicate postoperative outcomes [40, 41], we further analyzed the correlation between microcirculation status in the oral mucosa and surgical outcomes. Our results showed that patients with MRONJ who achieved complete mucosal healing after surgery had a statistically higher microvascular density in the oral mucosa than patients who did not. All four parameters, including TVD, PVD, PPV, and MFI, measured by HMV in healed patients were statistically higher than those in unhealed patients. Furthermore, we performed subgroup analyses for the ARM and ARM+AGM groups, respectively. Both groups were divided into subgroups according to the height of the four HVM microcirculatory parameters, and an univariate Cox regression analysis was performed. The results suggested that in the ARM group, the prognosis of MRONJ surgery was not correlated with the level of microcirculatory parameters, while in the ARM+AGM group, the prognosis of surgery in the “high” group with all four parameters was significantly better than that in the “low” group with all four parameters. The multifactorial Cox regression model further suggested that the level of TVD and PVD were the factors affecting the prognosis of surgery, and the prognosis of surgery was significantly more favorable in the “high” TVD or PVD group than in the “low” TVD or PVD group. Our findings suggest that AGM can aggravate the clinical state of MRONJ and surgical treatment outcomes by affecting mucosal microcirculation.

Antiangiogenic drugs are frequently utilized in targeted therapies for malignant tumors. While they offer anticancer benefits, these medications can also lead to adverse effects, particularly concerning the circulatory system and the kidneys [15]. Cardiotoxicity associated with the use of such therapies is mainly manifested by hypertension, thromboembolic episodes, and ischemic heart disease [42, 43, 44]. The most common renal complications include renal arterial hypertension, proteinuria, and microangiopathy [45, 46, 47]. The results of the present study tentatively suggest that long‐term application of antiangiogenic medications inhibits the healing capacity of oral mucosal wounds. Interestingly, the application of bevacizumab, a VEGF‐blocking drug, has recently been reported to cause delayed wound healing and reduce wound healing rates in patients with breast and ovarian cancer [48, 49]. Given the widespread use of such medications, oral mucosal healing disorders should be brought to the attention of oncologists, dentists, and patients undergoing antiangiogenic therapy. Antiangiogenic agents have been associated with diminished oral mucosal healing, thereby increasing susceptibility to MRONJ. Surgical treatment of MRONJ is challenging due to the impaired mucosal healing capacity, potentially leading to persistent osteonecrosis of the jaw. Conversely, the discontinuation of antiangiogenic drugs may compromise the treatment of primary malignancies, thus placing the management of such patients in a dilemma between antiangiogenic therapy and mitigating adverse outcomes.

Immunochemistry is a commonly used method for evaluating microvessels. However, this method requires the excision of mucosal specimens, which is invasive and requires a prolonged test period, making it unsuitable for clinical applications. High‐frequency Doppler ultrasound (HFD) and real‐time optical vascular imaging (RTOVI) were applied in previous studies to evaluate the status of oral microcirculation in patients with MRONJ [35, 37]. HFD quantifies blood flow velocities; however, it cannot image microvessel morphology and does not directly reflect the number of microvessels. RTOVI utilizes the high absorption of green light by erythrocytes to present microvessels and flowing erythrocytes in real time; however, the examination error is larger in the low‐perfusion region due to the reduction of erythrocytes in the low‐perfusion region. The introduction of HVM has allowed for direct visualization of the microcirculation of the skin, buccal mucosa, and sublingual mucosa [50, 51]. The principle of HVM based on side‐stream dark‐field (SDF) imaging technology lies in dark‐field microscopy, which employs oblique illumination. Only the light scattered by the sample is collected, while directly transmitted light is blocked, keeping the background dark. Microcirculatory structures can be observed in great detail: red blood cells are visualized as dark circulating bodies against a light background [52]. This technique allows for intuitive visualization of capillary density and red blood cell movement without being affected by the number of red blood cells inside the vessels. Characterized by easy operation, noninvasiveness, and rapid test results, HVM is appropriate for clinical applications, and accurate detection of oral mucosal microcirculation may predict the therapeutic effect of MRONJ treatment in advance. Therefore, it is expected that in the future, the appropriate timing may be selected according to the test results.

As part of the systemic peripheral circulation, the oral mucosa microcirculation status is closely related to blood pressure, and varying blood pressure in the population may affect the measurement results [53]. The optimal blood pressure for oral mucosal microcirculation measurements was explored to eliminate the effect of blood pressure on oral mucosal microcirculation. The above results indicate that when the blood pressure is MAPC (95–105 mmHg), the effect of blood pressure on microcirculation perfusion function and blood flow velocity (MFI) gradually reaches a peak plateau (PPV upper limit = 100%; MFI upper limit = 3), suggesting that an increase in blood pressure does not greatly affect microcirculation; therefore, MAPC was selected for the following study.

This study has several limitations. First, it had a small sample size and a relatively short follow‐up period. Second, although our findings offer a new perspective for assessing the healing capacity of soft tissues, the healing capacity of soft tissues is not only related to the density of microvessels or microcirculatory status but substantial factors, such as age and concomitant chemotherapy, may be involved in this process. Thus, large‐sample studies with longer follow‐up and deeper testing should be performed to further confirm these findings.

Our findings suggest that HMV can provide a non‐invasive and accurate measurement of the microcirculation status of the oral mucosa. Moreover, our data suggest that AMGs may worsen the disease condition of patients treated with ARMs and may further affect wound healing after surgical treatment.

## 4 Materials and Methods

### 4.1 Patients

This study is a bidirectional cohort study, including a retrospective MRONJ cohort, a healthy control cohort, and a prospective follow‐up cohort. A total of 30 MRONJ patients who underwent surgery at the Department of Oral and Maxillofacial Surgery of Peking University School and Hospital of Stomatology from February 2021 to August 2022 were included in this study. The inclusion criteria were as follows: (1) patients diagnosed with MRONJ stage 2 or 3 according to the American Association of Oral and Maxillofacial Surgeons 2022 guidelines [20]; (2) patients who agreed to undergo surgical treatment and those who did not undergo segmental osteotomy or free flap.

Based on their drug history, patients with MRONJ were divided into two groups: patients taking ARMs alone were assigned to the ARM group, and patients simultaneously taking ARM and AGM were assigned to the ARM+AGM group. Before the surgery, the responsible author YX. G. provided an introduction to the study content, surgical plan, and assessment indicators, as well as the objectives and significance of the research. Surgery was performed as previously described [54]; the surgical procedure included general anesthesia, radical osteotomy, and appropriate selection of soft tissue management to complete primary wound closure.

In addition, 15 age‐matched healthy volunteers with benign oral tumors were included in the control group. An oral mucosal tissue biopsy was collected from the margins of tumors that were not affected by the tumor. To reduce the influence of confounding factors on microcirculation, patients and healthy volunteers with uncontrolled hypertension, diabetes, or other diseases of blood vessels were excluded from our study. The process of participant enrollment can be referred to in Figure S1.

This study was approved by the Institutional Biomedicine Ethics Committee of the Peking University Hospital of Stomatology (PKUSSIRB‐202272011) on May 13, 2022. All the participants provided written informed consent. All the experimental procedures were performed in accordance with the principles of the Declaration of Helsinki.

### 4.2 Oral Mucosa Microcirculation Assessment

The mucosa of the floor of the mouth is thin, sufficiently elastic, and rich in blood flow, making it easy for the probe of the HVM to make complete contact with the mucosa and determine the exact microcirculatory status. Therefore, we chose the mucosa of the floor of the mouth as a representative site for the circulatory status of the oral mucosa. The oral mucosa microcirculation measurement was performed according to the following steps: (1) investigating the effect of different blood pressures on oral mucosa microcirculation in five healthy volunteers; (2) investigating the effect of different medication regimens on microcirculation in 30 MRONJ patients (15 in the ARM group; 15 in the ARM + AGM group) and 15 healthy subjects; (3) investigating the effect of different microcirculation statuses on wound healing in MRONJ patients at 1‐year follow‐up. Next, patients with MRONJ were divided into “healed” and “unhealed” groups according to their mucosal healing outcomes.

### 4.3 Blood Pressure Regulation

Anesthetic drugs controlled the patient's blood pressure; the mean arterial pressure (MAP) was used to describe the blood pressure levels. To ensure the safety of participants, the ranges of peripheral arterial blood pressure adopted in our study were set in three intervals: MAPA (mean arterial pressure A, 75–85 mmHg), MAPB (mean arterial pressure B, 85∼95 mmHg), and MAPC (mean arterial pressure C, 95–105 mmHg).

### 4.4 Detection of Oral Mucosal Microcirculation by HVM Device

**FIGURE 6:**
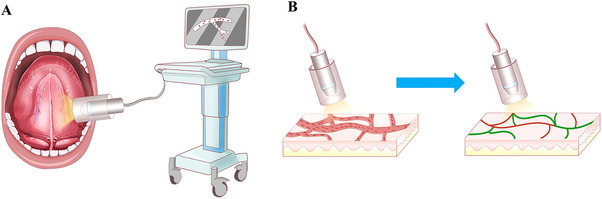
Schematic diagram for the measurement and analysis of sublingual microcirculation measurement by HMV. (A) The HMV probe was placed in direct contact with sublingual mucosa. After adjusting the focal length, a clear video of sublingual microcirculation was displayed on the screen connected to the probe. (B) A red curve represents perfused microvessels; a green curve represents sluggishly flowing or blocked microvessels. Four microcirculatory parameters were calculated according to the degree of patency of microvessels.

Microcirculation measurements were recorded (HY. H. and N. Z.), evaluated (N. Z.), and analyzed using a second‐generation HVM device (MicroSee, Guangzhou Yiruan Intelligent Technology Co., Ltd.) following the European Society of Intensive Care Medicine guidelines 2017 [27]. Briefly, saliva on the floor of the mouth was cleaned with dry gauze, and the HVM probe was placed in direct contact with the sublingual mucosa. After adjusting the focal length of the probe, steady videos lasting for at least 4 s were made (Figure 6A). Three random sites in the sublingual oral mucosa area that were not affected by MRONJ or tumor and could provide the sharpest microcirculatory images were selected. Sluggishly flowing or blocked flow was defined as the velocity or quantity of red blood cells below normal flow in participants under resting conditions. Four microvascular variables were calculated using the image analysis software built into MicroSee (Table S6; Figure 6B). The mean values of microvascular variables from the three sites were used for statistical analysis.

To determine the effect of different microcirculation indicators on the prognosis of MRONJ surgical treatment, we compared the four parameters of patients with the same medication regimen (ARM alone or ARM+AGM) according to their levels and average values. If the value of one microcirculation parameter was ≥ the median value of the ARM group or ARM+AGM group, it was assigned to the “high” subgroup; otherwise, it will be assigned to the “low” subgroup.

### 4.5 Detection of Oral Mucosal Microvessels by Immunohistochemical Staining

Oral mucosa tissues were obtained from the margin of MRONJ lesions or benign tumors, which appeared normal and were not affected by the lesion. The specimens were fixed with 4% paraformaldehyde (PFA) and embedded in paraffin. Consecutive sections were cut from the paraffin blocks and subjected to immunohistochemical staining for CD31 (zm0044, ZSGB‐BIO). After deparaffinization, hydration, and antigen retrieval, the sections were incubated with mouse anti‐human CD31 monoclonal antibody for staining. Histomorphometric analyses were performed in a blinded manner by two observers (HY. H. and JH. Z.) with two years of experience. Two researchers (HY. H. and JH. Z.) jointly reviewed the images and, after discussion, quantified microvessels and select fields based on the method modified from the procedure described by Weidner in 1995 [55]. Three areas with the highest density of blood vessels detected using CD31 were selected under a 40×field. Microvessels that manifested as single CD31‐positive endothelial cells or clusters of CD31‐positive endothelial cells were counted. The mean value of the three counts was defined as the MVD of the mucosal tissue.

### 4.6 Follow‐Up and Healing Evaluation

Follow‐up examinations were performed at five specific time points: 2 weeks, 1 month, 3 months, 6 months, and 1 year after surgery. The criteria for assessing postoperative wound healing were based on Ristow's research [56]. Complete mucosal coverage after the operation without signs of residual infection or exposed bone was considered complete healing.

### 4.7 Statistical Analysis

SPSS 24.0 (IBM Corp. IBM SPSS Statistics for Windows, Version 24.0. Armonk, NY) was used for statistical analysis. Assessment of data normality was conducted by the Shapiro–Wilk test. For the comparison of quantitative data between two groups, a Welch's *t*‐test is performed if the data follow a normal distribution; otherwise, a Mann–Whitney *U* test is used. If the data met normality and homogeneity of variances, one‐way ANOVA was performed, followed by Tukey's post hoc test. If normality was met but variances were unequal, Welch's ANOVA was used, with pairwise comparisons conducted via Welch's *t*‐test with Bonferroni correction. If the data did not follow a normal distribution, the Kruskal–Wallis *H* test was applied, followed by Mann–Whitney *U* tests with Bonferroni correction for pairwise comparisons.

Simple linear regression analysis was used to explore the correlation between the total vessel density (TVD) measured by the HVM device and MVD calculated through immunohistochemical staining. *p*‐value < 0.05 represented statistical significance. Statistical data are presented as mean ± SD. Univariate Cox regression was applied to analyze the factors influencing surgical prognosis, and independent variables with *p* < 0.20 were included for multifactorial Cox regression analysis. All statistical figures in this study were generated using R software (version 4.3.2).

## Author Contributions

Conceptualization and study design: Yuxing Guo and Chuanbin Guo. Data collection: Ning Zhao, Hongyuan Huang, Jianhua Zhu, and Ying Zhou. Data analysis: Ning Zhao, Qingxiang Li, Qiao Qiao, and Yuanning Yang. Writing – original draft: Hongyuan Huang, Ning Zhao, and Jianhua Zhu. Writing – review & editing: Yuxing Guo, Jianhua Zhu, and Qingxiang Li. Funding acquisition and project supervision: Chuanbin Guo, Yuxing Guo, and Jianhua Zhu. All authors have read and approved the final manuscript.

## Conflicts of Interest

The authors declare no conflicts of interest.

## Ethics Statement

This study was approved by the Institutional Biomedicine Ethics Committee of the Peking University Hospital of Stomatology (PKUSSIRB‐202272011) on May 13, 2022. All the participants provided written informed consent. All the experimental procedures were performed in accordance with the principles of the Declaration of Helsinki.

## Supporting information




**Table S1**: Demographic characteristics of 45 subjects enrolled in this study.
**Table S2**: Comparison of sublingual microcirculatory between the healing group and unhealing group (n = 30).
**Table S3**: The relationship between the microcirculation parameters with wound healing using univariate Cox regression analysis in ARM group (n = 15).
**Table S4**: The relationship between the variables with wound healing using multifactorial Cox regression analysis in MFI subgroup of ARM+AGM group (n = 15).
**Table S5**: The relationship between the variables with surgery prognosis using multifactorial Cox regression analysis in PPV subgroup of ARM+AGM group (n = 15).
**Table S6**: The definition of microcirculatory variables acquired by HMV.
**Figure S1**: Patient inclusion and exclusion process, as well as the timeline of patient care.

**Video S1**: Optimal blood pressure for monitoring sublingual microcirculation. (A‐C) Representative videos of the sublingual microcirculation during MAPA, MAPB, and MAPC. MAPA, mean arterial pressure A(7585 mmHg); MAPB, mean arterial pressure B(85~95 mmHg); MAPC, mean arterial pressure C(95105 mmHg).

**Video S2**: Antiangiogenic medication aggravates oral mucosal microcirculation in MRONJ patients treated with ARMs. (A‐C) Representative videos of sublingual microcirculation in the control, ARM, and ARM+AGM groups. ARM, antiresorptive medication; AGM, antiangiogenic medication.

**Video S3**: Antiangiogenic agents affect wound healing in MRONJ patients treated with ARMs and who underwent surgery. (A‐D) Representative videos of sublingual microcirculation include four subgroups: the high microcirculation subgroup and low microcirculation subgroup within the ARM group, as well as the high microcirculation subgroup and low microcirculation subgroup within the ARM+AGM group.

## Data Availability

The authors confirm that the data supporting the findings of this study are available in the article and its supplementary materials.
