# Supplementary material for: Antiangiogenic Medications Impede the Oral Mucosal Microcirculation and Interfere with Oral Wound Healing: A Complication Deserving of Attention
Source: MedComm (2020). 2025 Jul 11;6(7):e70279. doi: 10.1002/mco2.70279 (PMC12246555; doi:10.1002/mco2.70279)
Supplement: Supplementary file 1 — Table S1: Demographic characteristics of 45 subjects enrolled in this study. Table S2: Comparison of sublingual microcirculatory between the healing group and unhealing group (n = 30). Table S3: The relationship between the microcirculation parameters with wound healing using univariate Cox regression analysis in ARM group (n = 15). Table S4: The relationship between the variables with wound healing using multifactorial Cox regression analysis in MFI subgroup of ARM+AGM group (n = 15). Table S5: The relationship between the variables with surgery prognosis using multifactorial Cox regression analysis in PPV subgroup of ARM+AGM group (n = 15). Table S6: The definition of microcirculatory variables acquired by HMV. Figure S1: Patient inclusion and exclusion process, as well as the timeline of patient care. [file MCO2-6-e70279-s001.docx]

**Antiangiogenic medications impede the oral mucosal** **microcirculation and interfere with oral wound healing: a complication deserving of attention**

**Hongyuan Huang^1,2,3,4#^, Ning Zhao^1,2,3,4#^, Jianhua Zhu^1,2,3,4#^, Qingxiang Li^1,2,3,4^, Qiao Qiao^1,2,3,4^, Yuanning Yang^1,2,3,4^, Ying Zhou^1,2,3,4^, Chuanbin Guo^1,2,3,4*^, Yuxing Guo^1,2,3,4*^**

*^1^**Department of Oral and Maxillofacial Surgery, Peking University School and Hospital of Stomatology, Beijing, 100081, PR China*

*^2^National Clinical Research Center for Oral Diseases, Beijing, 100081, PR China*

*^3^National Engineering Laboratory for Digital and Material Technology of Stomatology, Beijing, 100081, PR China*

*^4^Beijing Key Laboratory of Digital Stomatology, Peking University School and Hospital of Stomatology, Beijing, 100081, PR China*

*# Three authors contributed equally to this work*

****Corresponding author:***

*Chuanbin Guo, E-mail: guodazuo@sinas.com, NO. 22, Zhongguancun South Street, Haidian District, Beijing 100081, China; Tel.: +86 10 62179977; Fax: +86 10 62173402*

*Yuxing Guo, E-mail:*[*gladiater1984@163.com*](mailto:gladiater1984@163.com)*, NO. 22, Zhongguancun South Street, Haidian District, Beijing 100081, China; Tel.: +86 10 62179977; Fax: +86 10 62173402*

**Table S1**. Demographic characteristics of 45 subjects enrolled in this study

|  | | Control  (n=15) | ARM  (n=15) | ARM+AGM  (n=15) | *p* | |
| --- | --- | --- | --- | --- | --- | --- |
| Average age ± SD(year） | | 57.6±11.80 | 65.13±13.90 | 59.53±8.54 | 0.24 |  |
| Gender | male | 7 | 7 | 11 | 0.23 |  |
|  | female | 8 | 8 | 4 |  |  |

**Table S1.** 45 subjects were enrolled in this study, including 15 healthy volunteers, 15 patients with MRONJ taking ARMs alone, and 15 patients with MRONJ talking to both ARMs and AGMs. There were no statistical differences among the three groups in terms of age and sex.  ARM, antiresorptive medication; AGM, antiangiogenic medication.

**Table S2.** Comparison of sublingual microcirculatory between the healing group and unhealing group (n = 30)

|  | Healing group | Unhealing group | Statistic | *p* |
| --- | --- | --- | --- | --- |
| TVD (mm/mm2) | 29.41±2.83 | 21.43±4.40 | t=4.727 | ＜0.001 |
| PVD (mm/mm2) | 26.09±3.36 | 15.71±5.59 | t=5.596 | ＜0.001 |
| PPV | 91.64±5.13% | 71.72±13.54% | U=197.0* | ＜0.001 |
| MFI | 2.5±0.33 | 1.89±0.39 | U=186.0* | ＜0.001 |

**Table S2.** The sublingual microcirculatory variables were acquired before the surgical treatment in 30 MRONJ patients, which were categorized into “healing” group and “unhealing” group based on follow-up data. Two-sided unpaired t-tests were used to compare TVD, PVD, PPV, and MFI between the “healing” group and the “unhealing group.” Data are presented as mean ± SD. TVD, total vessel density; PVD, perfused vessel density; PPV, proportion of perfused vessels; MFI, microvascular flow index.

**Table S3.** The relationship between the microcirculation parameters with wound healing using univariate Cox regression analysis in ARM group (n = 15).

| Microcirculation parameters (low vs high) | Hazard ratio | 95%CI | *P* |
| --- | --- | --- | --- |
| TVD | 1.069 | 0.066-17.118 | 0.962 |
| PVD | 0.935 | 0.0584-14.978 | 0.962 |
| PPV | 7.359E+07 | 0-NUM | 0.999 |
| MFI | 2.041E+09 | 0-NUM | 0.999 |

**Table S3.** CI, confidence interval, TVD, total vessel density; PVD, perfused vessel density; PPV, proportion of perfused vessels; MFI, microvascular flow index. NUM indicates the function error value.

**Table S4.** The relationship between the variables with wound healing using multifactorial Cox regression analysis in MFI subgroup of ARM+AGM group (n = 15).

| Variable | Hazard ratio | 95%CI | *P* |
| --- | --- | --- | --- |
| PPV (low vs high) | 3.645 | 0.755-17.598 | 0.107 |
| MRONJ stage (stage Ⅱ vs stage Ⅲ) | 1.851 | 0.277-12.379 | 0.525 |
| Duration of antiresorptive therapy (＜ median) vs (＞ median) | 0.675 | 0.167-2.736 | 0.582 |
| MRONJ location (maxilla vs mandible) | 0.652 | 0.120-3.553 | 0.621 |

**Table S4.** CI, confidence interval, PPV, proportion of perfused vessels. There were no statistical differences among these groups.

**Table S5.** The relationship between the variables with surgery prognosis using multifactorial Cox regression analysis in PPV subgroup of ARM+AGM group (n = 15).

| Variable | Hazard ratio | 95%CI | *P* |
| --- | --- | --- | --- |
| PPV (low vs high) | 1.610E+11 | 0-NUM | 0.998 |
| MRONJ stage (stage Ⅱ vs stage Ⅲ) | 1.485E-10 | 0-NUM | 0.999 |
| Duration of antiresorptive therapy (＜ median) vs (＞ median) | 0.252 | 0.050-1.277 | 0.096 |
| MRONJ location (maxilla vs mandible) | 1.283 | 0.240-6.849 | 0.77 |

**Table S5.** CI, confidence interval, MFI, microvascular flow index. NUM indicates the function error value.

There were no statistical differences among these groups.

**Table S6.**The definition of microcirculatory variables acquired by HMV

| Variable | Abbreviation | Definition | Units |
| --- | --- | --- | --- |
| Total vessel density | TVD | Total vessel length per the area of visual field photographed | mm/mm2 |
| Perfused vessel density | PVD | The length of perfused vessel per the area of visual field photographed | mm/mm2 |
| Proportion of perfused vessels | PPV | The radio of PVD to TVD | % |
| Microvascular flow index | MFI | Divide the entire visual field photographed into 16 parts and score every part (0=stop flow,1=intermittent flow,2=sluggish flow,3=normal flow), calculate their average | / |

**Supplementary Figure**

**
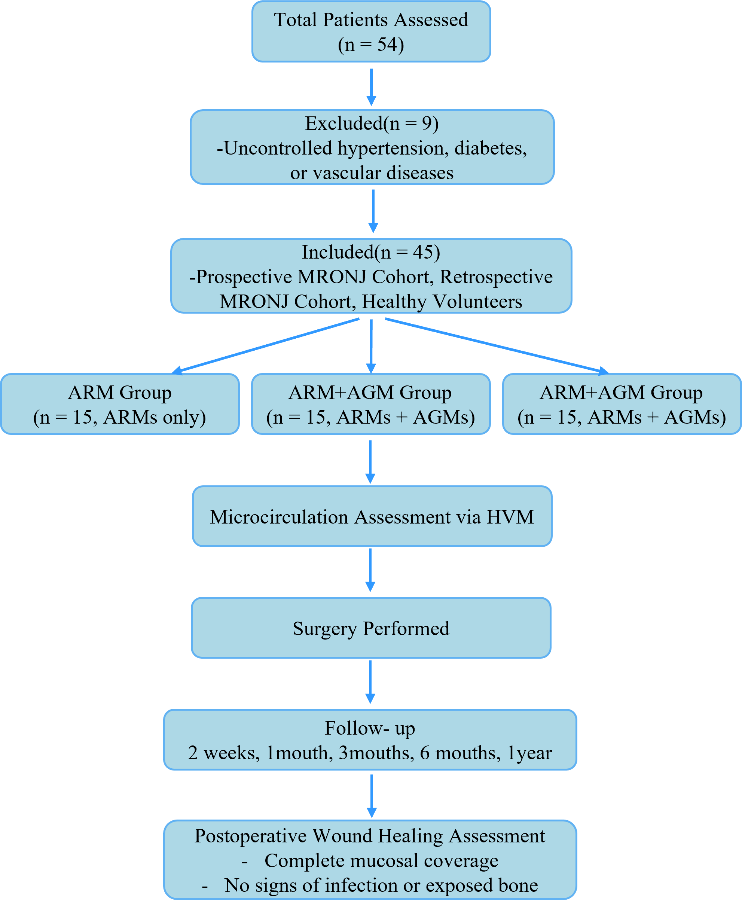
**

**Figure S1:** Patient inclusion and exclusion process, as well as the timeline of patient care.

**Supplementary Videos**

**Video S1: Optimal blood pressure for monitoring sublingual microcirculation. (A-C)**Representative videos of the sublingual microcirculation during MAPA, MAPB, and MAPC. MAPA, mean arterial pressure A(75–85 mmHg); MAPB, mean arterial pressure B(85~95 mmHg); MAPC, mean arterial pressure C(95–105 mmHg).

**Video S2: Antiangiogenic medication aggravates oral mucosal microcirculation in MRONJ patients treated with ARMs. (A-C)**Representative videos of sublingual microcirculation in the control, ARM, and ARM+AGM groups. ARM, antiresorptive medication; AGM, antiangiogenic medication.

**Video S3: Antiangiogenic agents affect wound healing in MRONJ patients treated with ARMs and who underwent surgery. (A-D)** Representative videos of sublingual microcirculation include four subgroups: the high microcirculation subgroup and low microcirculation subgroup within the ARM group, as well as the high microcirculation subgroup and low microcirculation subgroup within the ARM+AGM group.
